# Some Surprises in My Professional Career

**Published:** 1903-12

**Authors:** J. Paul Bush

**Affiliations:** Surgeon to the Bristol Royal Infirmary; Lecturer on Operative Surgery, University College, Bristol.


"*Scire est nescire, nisi id me*
*Scire alius sciret.*"

DECEMBER, 1903.

### SOME SURPRISES IN MY PROFESSIONAL CAREER.

BY

J. PAUL BUSH, C.M.G.,

*Surgeon to the Bristol Royal Infirmary;*
*Lecturer on Operative Surgery, University College, Bristol.*

**The Presidential Address delivered on October 14th, 1903, at the opening of the Thirtieth Session of the Bristol Medico-Chirurgical Society.**

A PRESIDENTIAL address may be of some use to our profession in the same way as a large river by its numerous tributaries brings down valuable soils from various geological formations, these become intimately blended in the stream of medical knowledge, and some grains may be deposited and bear fruit hereafter.

It is quite impossible for me, however, to give one of those learned or scientific addresses that have usually been delivered at this first meeting of the Session; I do not even intend to try to do this, and I shall be quite satisfied if any remarks of mine this evening add only a few drops to some small tributary running into the flood of useful knowledge, or even should a small portion of this address sink down as alluvial soil into the pages of the *Journal* of this Society.

As I have to follow so many eminent and eloquent presidents, some of whom I see here to-night, I shall have considerable difficulty I know in interesting you; but there is one thing I feel certain I shall have, and that is your profound sympathy on this occasion. You gentlemen who have already so ably presided over this Society know what anxiety you experienced when choosing a subject for your presidential address, and you gentlemen who will be filling this chair shortly must contemplate with awe the rapidly approaching day that is in store for you.

It was most difficult for me to select a subject for an address, but during the summer I prepared with much labour what I thought was a passable paper, entitled "The Uses and Abuses of Specialism." Having finished it, I thought it might be wise if I let some candid friend glance through it before its delivery, when to my surprise and horror he handed it back to me, saying, "My dear fellow, it won't do at all: you have put things in much too clear a light, and you would for ever offend some of your best friends." I thanked him for his trouble, and with a heavy heart threw my notes into the waste-paper basket.

A colleague suggested to me that I should address you this evening on "twenty years surgery at the Bristol Royal Infirmary"; but on commencing this subject, I found it would deal so largely with the figures of some four thousand major operations performed by myself, that I quickly gave it up as unsuitable.

My next idea was that as I had been Secretary of this Society for so many years, and knew the early history of its formation, that perhaps it might not be uninteresting if I recorded some of the discussions, scientific and otherwise, that have taken place at our ordinary meetings, and let you into some of the hidden secrets of the council chamber of the Society; but on second thoughts, I decided we were as yet too young to review our twenty-nine years of active work.

I propose, therefore, to relate to you "Some *surprises* I have met with in this the first portion of my professional career."

Some years before getting qualified I found myself, through no fault of my own, left in charge of a considerable practice just outside Bristol. During several months of this my summer holiday I had in connection with this practice to look after the wants of one of the large Bristol workhouses, containing some five hundred inmates. I visited daily with much pomp and vanity that institution, and being only a dresser at this period of my embryo existence, and commencing to learn the importance of Lister's new treatment of wounds, which was just commencing to be taught, I was much surprised to find that all the old bread poultices and other dirty healing applications had given place to an ointment, made in the union kitchen, of carefully sterilised dripping mixed with certain antiseptics, and which formed an ointment almost identical with the original Unguentum Acidi Boracis of Lister, and which certainly kept down the stink in the old wards and healed up the chronic ulcers like magic. Another little point that surprised my simple mind during my work at this union was the case of pauper "X," who was an inmate off and on for several years. He had a cunning way of keeping himself supplied with various kinds of luxuries. He used to write to all sorts of firms for samples, and obtain usually by return of post quite large quantities of tea, sugar, wine, spirits, tobacco, and even cigars, addressed to "So-and-So, Esq., So-and-So Workhouse." Evidently he was thought to be the Chairman of the Board, or the Master at least. This went on for some time, but he was at last found out by one of the firms writing to the Chairman and asking why they had never received an order for goods after sending so many samples.

When at work at the Royal Albert Hospital, Devonport, we received a great shock—and it must have been a most unwelcome surprise to the public generally, and to the medical profession and those whose duty it was to look after the national health in particular—when the Radical majority in Parliament, pandering to the misguided whims of cranks and other faddists, in 1881 refused, almost without a warning, to grant the usual funds for carrying out the "Contagious Diseases Act," and consequently some eighty beds had to be closed in Plymouth alone, and thousands of infected women throughout the country were allowed to spread broadcast and unrestricted the fell disease of syphilis and other venereal complaints. What this meant to the health and strength of the army and navy can never properly be realised; ay, and not only did this evil affect the bodies and life of our national defenders, but it penetrated deeply into the families and homes of thousands of innocent men and women far away from the garrison towns in which this Act had hitherto been applied; henceforth all chance of healing, both morally as well as physically, these fallen women was lost.

Another surprise was when I found myself in 1882 commencing to do a term in the convict prison at Portland. At this time there were over 1600 of the worst criminals in England, all undergoing long sentences of hard labour. Their wants and requirements were attended to by some 260 officers of the prison. The result of my first operation there was a surprise to me, and one not likely to occur again, as an empyema of both sides was completely cured by aspiration without drainage.

It was surprising to me, as a very young man, to see the ingenuity and power of ready invention shown by some of the prisoners in feigning diseases; and I could recount to you in detail numerous clever devices of malingering which were detected, if I were not prevented by the Official Secrets Act.

The educated criminals were the class which produced the most difficult cases of this nature: for instance, one prisoner who had a life sentence gave us much trouble in unravelling his counterfeit symptoms of a severe nerve lesion: and another took us in for a time more than once, for it was several days before we discovered that his fictitious hæmoptysis was manufactured by his scratching the posterior surface of a large tonsil with his nail, and that he was mixing the hemorrhage thus caused with saliva in his chamber utensil.

Artificially induced vomiting and diarrhœa, from picking up and eating when out on the works various little items not intended for human food, had to be carefully looked for; and it was surprising how easily some people could bring down a hernia, even when wearing a properly fitting truss.

I met with several surprises during the years 1899 and 1900, some of which may interest you if I mention them.

The Boer ultimatum reached England October 10th, 1899; and most of us, I think, were surprised when we found ourselves engaged in a war that was not brought to a finish by the end of that year. Our early reverses in South Africa kindled the military spirit that permeates all Englishmen, whether they be fighting men by profession or civilians, and in November I found myself with a host of others volunteering for active service. But owing to difficulties which arose in connection with one of my public appointments in Bristol, I was unable to proceed to South Africa until a little later, when I was asked to take out the Princess Christian Hospital, a hospital provided and maintained by the generosity of Mr. Alfred Mosely, an old Bristol man.

In making arrangements for a hospital which was to be about the same size as the Bristol Royal Infirmary many surprises were in store for me; and I wondered at the huge number and variety of things required for hospital equipment, ranging from cows to safety pins, and from house-flannels to X-ray apparatus.

For transport reasons our equipment had to be kept down as low as possible, and to diminish the size of the drug list I asked my five surgeons to each send me a list of the preparations they were in the habit of ordering most frequently. I was surprised to find that the combined list contained almost every preparation in the *Pharmacopœia*, together with some drugs I had never heard of; with the help of a fountain-pen, during a journey to London, I was able to reduce the list to one-twentieth of its original size.

The same thing happened with regard to medical books which one of my officers wished to take out with him as hospital stores; such works as *Donders on Refraction*, *Van der Kolk on the Medulla*, various treatises on diseases of children, tumours in connection with the pregnant uterus, and such-like volumes had to be left behind.

We sailed in February, 1900; and when we got on board we were surprised to find that our hospital staff had to commence work at once, whether suffering from sea-sickness or not, as we had some three thousand souls on the ship to look after professionally.

On arriving at Cape Town we were much surprised to hear that Ladysmith had been relieved, and that the war was not over yet. The hospital and ourselves were ordered at once to Bloemfontein ; but as soon as we had got most of our belongings off the ship we were instructed that our services were required in Natal, and so we re-embarked and proceeded to join Buller's army.

After selecting the ground and doing a certain amount of levelling, it was surprising how rapidly our buildings went up. It was indeed a surprise when we discovered that an ordinary "take-in day" meant the admission of about one hundred new cases, all seriously ill. And it may surprise some of you physicians when I tell you that sometimes we were unable to say whether a case was enteric or dysentery ; even a *post-mortem* examination did not always clear up our doubts on this point, and I am inclined to think that very frequently the patient had both diseases.

Of all the fell enemies with which the British had to deal, undoubtedly the bacillus typhosus was the most deadly. Unfortunately our intelligence department, if I may apply the term to our bacteriological research laboratories, had not got its information sufficiently complete to enable us to check the inroads of this invader ; and the result is most surprising, as we are left after this long and deadly war with unreliable data upon which to declare our verdict for or against the value of antityphoid serum with any degree of certainty.

These results might have been different had it been known then, as it is now, that the immediate effect of inoculation is to produce a phase, varying in length from one to many days, during which the patient is actually more susceptible to the disease than he was before inoculation. This being the case, it is natural that an infected area, such as a camp, should be the very worst possible place in which to carry out the procedure, and good illustrations of this occurred in Ladysmith and other districts.

The selection of the serum seems to require great care, and different sera varied considerably in their immediate effect. Some surgeons surprised me much when they told me that they had seen scarcely any symptoms follow the injection; but with us on board the troopship *Assaye* nearly every patient injected developed some temperature, and many became delirious. I myself, after receiving my first injection, performed the operation on several hundred others, but had to knock off in the afternoon with a temperature of over 103°; and my juniors informed me that for twenty-four hours I talked a great deal more nonsense than usual. I have no recollection of this, however, and you must take this statement for what it is worth; but I do know that for a couple of days I did not feel fit for any work at all.

Taking some 90,000 patients who were the subjects of typhoid vaccination during 1899 and 1900, we find that the inoculated men had only half the susceptibility to infection of that shown by the uninoculated, and the proportion of deaths among the inoculated was only half that among the uninoculated; therefore from the numbers it appears that inoculation for typhoid has done something to diminish the number of deaths from enteric fever, and with more definite sera and improved methods of inoculation we may reasonably hope for still better results before long. It is surprising to hear that the army authorities are proposing to discontinue the systematic inoculation of troops. I trust not.

Most of our typhoids came into hospital in the third week of their illness; and the anxiety of these cases was not lightened by the fact that a great deal of the nursing had to be done by the St. John's Ambulance orderlies—an excellent set of men in their way. And it was surprising how well they did, considering they had had no previous experience of hospital nursing until they learnt something on the troopship going out. They turned out a good lot, and willing to be taught the elementaries of tending the sick by our six most excellent nursing sisters. I cannot help mentioning the following which occurred during our first week. A night orderly failed to report a hemorrhage of about a pint from an enteric patient, because it was, he said, only *venous*

blood, and therefore not of any importance! We soon found out that it was necessary for the medical officer on duty to make numerous night visits to the wards, as some of the orderlies' night reports were of doubtful value, especially when they contained such statements as this: "Patient had his bowels *re*moved twice during the night." The author of this blunder, when spoken to on this subject, said: "But, doctor, permit me to vindicate my official position." The medical officer wisely moved on to the next patient, and left him vindicating his official position. One of our orderlies, an Irishman, was at first inclined to be a bit independent; and on a certain morning round appeared late in the ward, and excused himself by saying that "the Irish mail was in."

Another danger, apparently inseparable from inoculation, was the false sense of security which it established among some of the troops. I frequently heard those who had submitted to the operation chaffingly tell the "passive resisters" that they would be sorry when they would some day come "panting to the pool," but would be afraid to drink because they were not proof against enteric, thereby implying that they themselves, having been immunized, would have no compunction about drinking even infected water.

Although every precaution is taken in a camp or hospital to disinfect excreta and clothes—enormous quantities of corrosive sublimate and lime being used—still there is room for improvement in the sterilizing of water. We found that it was only by means of padlocks that the Kaffirs and Coolies employed in the hospital could be prevented from dipping their filthy hands and dirty bowls into the storage tanks of drinking water; and where these tanks had to be uncovered in places, they had actually to be guarded by a sentry to prevent pollution by our own people.

I am surprised that the authorities have not universally adopted some of the numerous patterns of field water-carts that have been suggested over and over again, constructed in such a way that at any moment the entire contents could be easily boiled, and so ensure a fair amount of sterile water being always available at the shortest notice.

In South Africa I am quite aware of the fact that frequently there was no fuel of any kind to be found; and I hope the authorities will—if they have not already done so—consider the question of the use of petroleum, as it is stated that an ounce of this will produce enough heat to boil sufficient water for a man's use for twenty-four hours.

It is without question, I think, that the water supply was the main source of the spread of the infection of enteric fever in South Africa; but we must not take it for granted that if this liquid is made sterile and kept free from contamination, that then we are safe from the ravages of this fearful disease. We do not yet know what amount of enteric is produced by the awful dust storms that all our hospitals had to encounter; and it was surprising to notice how rapidly the myriads of flies were produced when the bad cases began to arrive: how much this mode of inter-communication had to do with the spread of disease, and how to deal with the plague of flies, we are at a loss to know. The other great plague—I mean the plague of women, that was worse than the flies in some of the hospitals—I am thankful to say we were spared, as we were too far from any large town for them to come in any numbers, and there was no place near for them to stay. We had a few residents who lived close to the hospital, and who were of very great assistance to us in supplying fresh fruit and flowers.

It was very surprising how comfortable our wooden huts were made; but there was always an anxiety about fire, as not only did we allow the patients to smoke in the wards, but during the hot weather we had to be on the look-out for bush fires, which were frequent and extensive round about us.

It was astonishing to me how our native servants made us "fire proof" by firing the dry grass and undergrowth close up to our buildings, and then driving the flames towards the approaching danger.

Our Zulu labourers were an interesting study, and we learnt something of their manners and customs while we were up country in Africa. It surprised us to hear that a Zulu woman never called her husband by his name, either when speaking of him to us or when addressing her spouse: she always used the phrase "Father of So-and-So." The husband had a peculiar haughty contempt—if I may put it in that way—for his wife, for if they both were going in the same direction they would not walk together. At the same time the Zulus had very decided views concerning the ill-treatment of their women. For instance, it was surprising to learn that the wife could always depart to the home of her father if her husband was brutal to her, and there she stayed till her better half had expiated the offence. The *modus operandi* was this. He had to go in person and ask for his wife again. On his arrival at her village he was instantly set upon by all the women, young and old; and they had most likely been waiting a day or two for this hour of vengeance to arrive. By their laws they might not only use their tongues, but their nails and fists as well. Nor was the unfortunate man permitted to protect himself in any way. Having suffered much, he was not even then allowed to take his wife home again; but he had to return to his location with the sentence that he must pay over to his father-in-law a certain number of cows before he could demand his wife: the market value of a wife ranged, according to her weight and fatness, from five to ten cows.

We had a very complete set of instruments for doing X-ray work; and we found it was most convenient having this department in a room leading out of the operating theatre, with a dark room adjoining, so that plates could be developed and shown at once: consequently a large number of intricate injuries were sent to us for reports.

On visiting one of the big hospitals in South Africa I was much surprised and somewhat amused at hearing that the whole of their first supply of skiagraphic plates had been spoilt by being stored in the same hut where the X-ray apparatus was being worked.

On my return to England it was surprising, but at the same time most annoying, to hear grumblers, sitting in their armchairs at home, or in front of a comfortable fire at their club, and some also on political platforms, complaining in no uncertain terms of the hospital arrangements generally; and some of the remarks of a certain feather-bed critic, assisted by his anonymous friend the eminent but pugilistic Harley Street consultant, were in my humble opinion quite uncalled for. Some of these irresponsible and ignorant critics, eaten up perhaps with the idea of some self-advertisement, whose sympathy for our sick and wounded appeared to take the form of almost malicious complainings of the deeds of others, rather than of aiding the nation by some substantial help, did not seem to understand that the first duty of a country at war with another is to fight; hence hospital stores, etc., which at times I admit were badly needed, had to stand aside, while men, ammunition and food were hurried forward over the single line of rails leading to the front. It is the old story of the necessity of the bullets having the preference of the pills. During 1900 I visited nearly every large hospital in South Africa, and saw something of their working; and I make the statement that although, of course, there were mistakes made, no army has ever been so well supplied with hospitals and all their comforts as our men were in South Africa.

On looking up the details of the Crimean War, I am surprised to find that if we had lost the same per centage in South Africa as we did in the Crimea, we should have lost 75,000 men; but our loss was about 25,000, and so we gained 50,000—if I may put it in that way.

The Royal Army Medical Corps did splendid work; their deficiencies, through no fault of theirs, were in numbers only, as they were about 25 per cent. short of their proper strength for an army of the size with which we commenced the Boer War. And as the war proceeded the Medical Department was called upon to provide officers for an army three or four times the size of that for which its establishment had been estimated as sufficient. And if in the time of peace the British taxpayer will not pay for a sufficiently strong Army Medical Department, the necessity must arise for seeking help from civilians; and if we ever have another great war—and I am not one of those who imagine we are going to live in peace evermore—the Royal Army Medical Corps must depend on at least 40 per cent. of the medicals being civil surgeons; but this assistance of the civilians must be organized and paid for, and the sooner the better.

It surprised me to hear on my return that in some hospitals the civil and military elements did not quite hit it off. All I can say is that in my own case, and I believe also in the case of the excellent civil surgeons I took out with me, that there never was one single word of discord between us and my friend, Major Mathias, D.S.O., who was the military man attached to us, and who was the responsible officer for the numerous War Office reports, evidently drawn up by men accustomed to use an excessive amount of red tape. He was a first-rate fellow, and one who did everything to prevent friction of any kind. We were the only hospital run entirely by civilians in Natal; and I think it has been a surprise to the country in general how well this new idea of having civil hospitals attached to an army in war time worked out. It is an experiment that is likely to be followed in the future.

On looking up my books, it surprises me to find I have been examining recruits for the Bristol Police Force for twenty years; and during that period I find I have examined just under two thousand men for our Bristol force, besides a large number for the Colonial and Government services.

Candidates not attaining to our high standard in height and chest measurement are weeded out before coming to me, and all have to bring a medical certificate of fitness for police work.

It surprises me to find that I have been able to pass as fit only some 40 per cent. of these. A flattening of the plantar arch, commencing varicose veins, and defective hearing are the commonest causes of rejection; and in spite of this being the case, a large number of the men I have had to discharge from the force before they have served long enough for a pension, have been incapacitated by varicose veins or flat feet.

For many years I have given up rejecting men on account of a moderate varicocele only; and I have not had a single example of this condition giving trouble afterwards. I have been much struck with the small number of cases of phthisis occurring in policemen. Is this due to the open-air life they lead? Certainly, from their appearance, they feed themselves well!

It has surprised me to find that country men, and even country policemen, on joining the town force complain a great deal of their feet and legs giving out. Is this due to the stone pavements or to the wooden roadways, or possibly to the area steps so frequently found in the cities.

In going over my notes of hernia cases, I am surprised to find what a large number of patients had a hernia of the large intestine. The appendix, as everyone knows, is frequently found in a hernial sac; but it surprised me a good deal a short time ago when I discovered that fashionable appendage sloughing in the scrotum. And it has not happened to everyone, I think, to have removed about a third of the urinary bladder without apparent discomfort to the patient, but with much surprise and regret to myself, in a case of a large omental hernia which completely surrounded and hid the herniated bladder. Several other unusual contents I have found in hernial sacs. On one occasion I unearthed a strangulated ovary and Fallopian tube in an inguinal hernia; and in another I discovered intestine completely blocked by a large gall stone; and in another strangulated hernia there was a cancer of the small intestine which I was able to remove successfully.

On looking up the subject of the removal of the ovary for disease, I am surprised to find it has fallen to my lot to have performed a successful ovariotomy on the oldest patient yet recorded, namely in a woman of 85, from whom I removed a cyst containing 19 pints. And I am inclined to think that the child, aged 6 months, from whom I removed a diseased ovary is one of the youngest patients that have lived to tell the tale.

About six years ago I first saw a young unmarried woman with a large, hard, irregular swelling below the liver. An exploratory operation revealed an irremovable mass which seemed obviously to be malignant. Some years later, as her condition remained the same, I again explored, but now I found the glands everywhere infected with what was considered, after a microscopic examination, to be sarcoma; and I had to abandon the operation for the second time. This year she again came into the Infirmary on account of the increase in size of the tumour. She was no worse in her bodily health, but complained of very great and increasing pain in the abdomen. Thinking that after the lapse of so long a time the diagnosis must be at fault, for the third time I explored the belly cavity, and while poking about and trying to separate adherent intestines from the tumour, some stuff, looking like butter, came away in large quantities, then some very hard things like nail heads were felt, and after that a tooth or two came into view at the bottom of the pit. During the operation it was also suggested it might be a case of extra-uterine pregnancy, or even a cancer of the pancreas. Eventually, after coming across a fœtal skull, a tongue or two, and some other curiosities in the way of hair and fingers, I removed a teratoma from beneath the liver. She has made an excellent recovery.

Not very long ago I saw in consultation a case at one of our big lunatic asylums that looked very like a perforated gastric ulcer. She was very ill, but still insisted that she had several devils inside her. This, of course, we considered was one of her delusions; but when we extracted, by means of an abdominal section, an equal number of long hat pins from her stomach and peritoneal cavity, we came to the conclusion that it was not so much of a delusion after all; and we were surprised to find that, having got rid of her real devils, she recovered mentally as well as physically.

It has more than once surprised me how much septic material the peritoneal cavity will sometimes put up with. The doctor of the case will I know excuse my mentioning the following, which happened in private to me only a few months ago. I was operating in the middle of the night, and somewhat short-handed, on a large abdominal swelling, when suddenly the patient ceased breathing and appeared to be dead. Artificial respiration had to be performed for some time, during which several coils of intestine escaped on to the table, which had become greatly disarranged in regard to the antiseptic surroundings. We had no time even to wash the intestines before reducing them on account of the patient's struggles; and while we were doing this the patient involuntary inserted her hand into her own peritoneal cavity. She, however, made a first-rate recovery, and was none the worse for the unique experience of having herself handled her own internal organs without first washing her hands.

The following surprising case shows what some people will stand. Some years ago a mass, which had once been an engineer's assistant, was brought into the casualty room at the Infirmary. He had got caught on the shaft of a large machine, and was rotated on it at full speed for nearly three minutes. During his evolution his legs smashed through a window close to a workman in the next room, and so frightened him that he has never been the same man since. On going carefully over the case, this apparently lifeless mass was found to be a boy about 16 years of age, with seventeen fractures of major bones, including a fractured base of the skull and numerous other injuries. After a long illness he became convalescent, and received a considerable sum of money for his injuries; he is now one of the engineers of the Royal Infirmary, and apparently none the worse for his numerous fractures.

In conclusion, gentlemen, I must thank you for the very patient way you have listened to these very unscientific remarks of mine, and for the honour you have done me in placing me in your President's chair for the ensuing year.

